# Neural correlates of behavioural symptoms in behavioural variant frontotemporal dementia and Alzheimer's disease: Employment of a visual MRI rating scale

**DOI:** 10.1590/S1980-57642012DN06010003

**Published:** 2012

**Authors:** Christopher Go, Eneida Mioshi, Belinda Yew, John R Hodges, Michael Hornberger

**Affiliations:** 1 Neuroscience Research Australia, Sydney, Australia.; 2 Faculty of Medicine, University of New South Wales, Sydney, Australia.

**Keywords:** behavioural symptoms, apathy, magnetic resonance imaging, behavioural variant frontotemporal dementia, Alzheimer's disease

## Abstract

Frontotemporal dementia (FTD) patients often present with severe behavioural disturbances and concomitant lack of insight. The underlying neural correlates of these disturbances are mostly attributed to prefrontal cortex dysfunction, but are still poorly understood.

**Objectives:**

The current study explores whether a simple visual magnetic resonance imaging (MRI) rating scale in combination with the Frontal System Behaviour Scale (FrSBe) can be used to identify the prefrontal correlates of behavioural symptoms in behavioural variant frontotemporal dementia (bvFTD) and Alzheimer's disease (AD).

**Methods:**

Forty-eight patients with a clinical diagnosis of bvFTD and AD participated in the study. Their behavioural profiles were assessed using the Frontal System Behaviour Scale (FrSBe) and cross-correlated to the atrophy of the sub-regions in the prefrontal cortex using a 5-point visual rating scale of MRI scans.

**Results:**

Patients with bvFTD showed higher incidence of behavioural disturbances than AD with apathy being the most significant. BvFTD patients also showed the highest incidence of atrophy in the orbital frontal cortex and this atrophy was correlated with the apathetic features.

**Conclusions:**

Employment of a simple visual MRI rating scale can be used in combination with a behavioural screening test to identify reliably the behavioural symptoms in bvFTD and AD. These findings will inform the diagnostic accuracy of the neural correlates of behavioural dysfunction in bvFTD in the future.

## Abstract (pt)

Pacientes com demência frontotemporal (DFT) frequentemente se apresentam com graves distúrbios comportamentais e concomitante falta de *insight*. Os correlatos neurais subjacentes a estes distúrbios são em sua maioria atribuídos a disfunção do córtex pré-frontal, porém, ainda são pouco compreendidos.

**Objetivos:**

O presente estudo explora se uma escala de mensuração visual de ressonância magnética (RM) em combinação com a Escala Comportamental do Sistema Frontal podem ser usadas para identificar os correlatos pré-frontais de sintomas comportamentais na variante comportamental da DFT (cDFT) e na doença de Alzheimer (DA).

**Métodos:**

Quarenta e oito pacientes com diagnóstico clínico de cDFT e DA participaram do estudo. Seus perfis comportamentais foram avaliados usando a Escala Comportamental do Sistema Frontal (ECSF) e correlacionada a atrofia das sub-regiões no córtex pré-frontal utilizando uma escala de mensuração visual de 5 pontos na RM.

**Resultados:**

Os pacientes com cDFT mostraram uma maior incidência de distúrbios comportamentais do que os com DA, sendo a apatia o sintoma mais significativo. Os pacientes com cDFT também demonstraram uma maior incidência de atrofia no córtex orbito-frontal e esta atrofia correlacionou-se às características apáticas.

**Conclusões:**

O emprego de uma escala simples de mensuração visual de RM pode ser usada em combinação a um teste de rastreio comportamental para identificar de forma confiável os sintomas comportamentais na cDFT e DA. Estes achados informarão a acurácia diagnóstica dos correlatos neurais da disfunção comportamental na cDFT no futuro.

## INTRODUCTION

Frontotemporal dementia (FTD) is the most common early onset dementia^1^ after Alzheimer's disease. At present, however, the diagnosis of FTD patients remains challenging, in particular for the behavioural symptoms in the patients, which can overlap with other neurodegenerative conditions, in particular Alzheimer's disease (AD) but also psychiatric diseases, such as schizophrenia.^2^ Correct identification of behavioural disturbances relies on experienced clinicians and perceptive carers to elicit an accurate behavioural profile,^3^ in particular because FTD patients show usually loss of insight. More formal carer assessments of the patients' behaviours (e.g. Neuropsychiatric Inventory - NPI),^4^ Cambridge Behavioural Inventory - CBI^5^ have also been used to characterise the behavioural symptoms [e.g.].^6^ The neural correlates of these behavioural symptoms have only been recently explored and show that atrophy in prefrontal cortex regions is to a large degree responsible for the behavioural disturbances.^7,8^ Similarly, Peters et al.^9^ found that apathy and disinhibition scores were related to ventromedial prefrontal cortex dysfunction in FTD.

A more recently developed tool, the Frontal System Behaviour Scale (FrSBe) takes a slightly different approach to the NPI and CBI by asking for any premorbid symptoms before disease onset as well as any current symptoms. The FrSBe also takes into account the patients' perspectives as well, by allowing a symptom self-assessment by the patient which can be contrasted to the carer assessment. Further, the FrSBe focuses in depth on three behavioural symptoms areas: apathy, disinhibition and executive dysfunction which are all dominant disturbances in FTD patients.^10,11^ A recent study,^12^ employing the FrSBe in FTD patients, found that both behavioural and language FTD patients scored high on all FrSBe scores, indicating that both groups experience behavioural disturbances. Crucially, both groups (behavioural vs. language) differed only on the disinhibition subscore of the FrSBe. A voxel-based morphometry analysis found a correlation between atrophy in prefrontal and temporal cortex regions and the severity of the apathy and disinhibition FrSBe subscores. Nevertheless, all previous FrSBe studies in FTD did not take into account the patient's own evaluation of their symptoms. Further, VBM analysis are not feasible to perform in a clinical setting and therefore it is unclear whether simple visual rating atrophy scales can also be used to relate the behavioural dysfunction to the underlying neural correlates.

The current study explored the behavioural dysfunction in sample of behavioural variant frontotemporal dementia (bvFTD) patients, who show the most significant behavioural changes in the FTD spectrum, by

(i) contrasting the pre- and post-disease symptom assessments of bvFTD patients and their carers;
(ii) employing a visual rating scale of the patients MRI scans to relate their symptoms to the underlying atrophy; and
(iii) contrasting the bvFTD patients against an AD patient cohort.

We hypothesised, that bvFTD patients would show lower concordance with their carers on reported symptoms than AD patients. We further predicted that ventromedial prefrontal cortex atrophy would be most severe in bvFTD and would correlate with severity of disinhibition and apathy.

## METHODS

**Case selection.** Patients were consecutively selected from the FRONTIER Dementia Clinic Database, resulting in a sample of 30 bvFTD and 18 AD patients. All patients included were assessed and scanned at the first clinic visit. All FTD patients met the current consensus^13,14^ for FTD with insidious onset, decline in social behavior and personal conduct, emotional blunting and loss of insight while AD patients met NINCDS-ADRDA diagnostic criteria for probable AD^15^ All patients and caregivers completed the FrSBe to assess the behavioural symptoms. The study was approved by the University of New South Wales Human Research Ethics Advisory panel D (Biomedial, ref. #10035).

All patients were assessed comprehensively through a multidisciplinary approach through a combination of the senior neurologist (JRH) clinical report on presentation, neuropsychological assessment, structural neuroimaging, as well as the carer's assessment of patient's behaviours.

Disease severity was determined using the Frontotemporal Dementia Rating Scale (FRS).^16^ The FRS yields 6 different disease stages, ranging from very mild to profound, on the basis of changes in activities of daily living and behavior. The range of dementia stages for the FRS Rasch score are very mild (5.39 to 4.12); mild (3.35 to 1.92); moderate (1.68 to -0.40); severe (-0.59 to -2.58); very severe (-3.09 to -4.99); and profound (-4.98 to -6.66). The FRS Rasch score is obtained through an interview with the caregiver or the proxy informant.

**Test selection.** The FrSBe was used to assess the behavioural disturbances of the patients. This is a 46 item rating scale that accesses the function of the frontal lobes and compares the behaviours of the patients before and after illness onset using the 5-point Likert scale and is completed by both the patients and their informants.^17^ It measures behavioural changes in 3 areas: Apathy, Disinhibition and Executive Functions. The score of the patient is then collected and compared against normative data on 436 healthy adults. If the score obtained is higher than the baseline results, it is indicative of frontal lobes damage.^18^

The Addenbrooke's Cognitive Examination Revised (ACE-R) and Cambridge Behavioural Inventory (CBI) were also used to assess the patients. ACE-R is a 100 point evaluation that assesses 5 cognitive domains: attention/orientation, memory, fluency, language and visuospatial^19^ while CBI is an 81 item questionnaire that assesses cognitive, behavioural and affective symptoms as well as activities of daily living and evaluates various functional/behavioural domains using a 5 point rating scale.^20^

*Image Acquisition & analysis:* All patients underwent the same imaging protocol with a whole-brain T1-weighted images using a 3-tesla Philips MRI scanner with standard quadrature head coil (coronal orientation, matrix 256 × 256, 200 slices, 1 × 1 mm^2^ in-plane resolution, slice thickness 1 mm, TE/TR=2.6/5.8 ms, flip angle a =19º).

**Figure 1:**
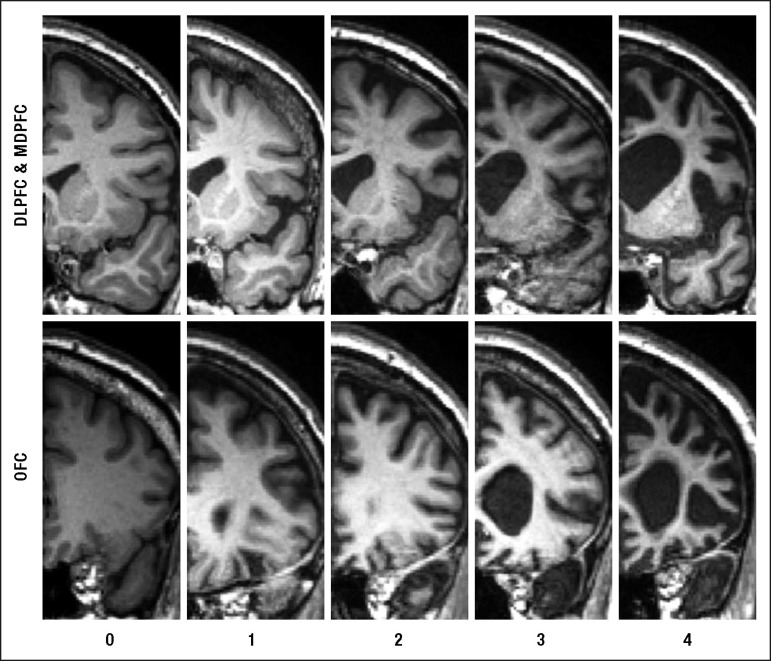
Shows the array of MRI reference images and rating criteria employed in judging atrophy in the frontal lobe brain regions. Rating criteria range from 0 = no atrophy to 4 = severe atrophy for the three prefrontal brain regions (OFC: orbitofrontal cortex; MPFC: mesial prefrontal cortex; DLPFC: dorsolateral prefrontal cortex).

One rater (CG), blind to the clinical diagnosis, rated T1 coronal MRIs based on a visual rating scale developed by Davies and colleagues^21-23^ using a standard template against which to judge atrophy. The rater showed high reliability for the scoring of a MRI training set of 100 scans (Cronbach alpha=0.95). In brief, the rating method involved assessments of two coronal slices: the first at the level of the anterior temporal pole and the second at the level of the insula. More detailed description of the rating method can be found elsewhere.^22,23^ Four prefrontal regions were scored: orbital (OFC), medial (MFC), dorsolateral (DLPFC) and total prefrontal cortices (PFC). Atrophy within each region was rated on a 5-point Likert scale ranging from 0 to 4 (0=normal; 4=severe atrophy) (Figure 1). The orbitofrontal region was rated on the coronal image where the anterior temporal pole is first visible. Medial and dorsolateral frontal regions were rated on the second coronal slice. This image was the most posterior slice through the temporal pole without visible connection between frontal and temporal lobes (Figure 1). The total prefrontal atrophy was obtained by adding the atrophy ratings from the other 3 sub-regions.

**Statistics.** Data were analysed using SPSS 18.0 (SPSS Inc., Chicago, Ill., USA). Parametric demographic (age, education), neuropsychological (general cognitive tests), behavioural (FrSBe, CBI) and scan ratings (MRI) data were compared across the 2 groups (bvFTD, AD) via repeated measure ANOVAs Greenhouse-Geisser corrected, as well as independent t-tests. Sex differences were assessed via a Chi-square test. A priori, variables were plotted and checked for normality of distribution by Kolmogorov Smirnov tests. Variables revealing non-normal distributions were log transformed and the appropriate log values were used in the analyses.

## RESULTS

**Demographic and background analysis**. Comparisons across the two groups revealed no significant difference for the demographic variables of age, education and sex (all p's>0.1) (Table 1). Further analyses showed significant group effects for the CBI [F(1.44)=10.3, p<0.01] and Rasch Score [F(1.30)=5.13, p<0.05] (Table 1). Due to the lower Rasch scores in the bvFTD group, we included the Rasch scores as a covariate in the remaining analyses.

**Table 1:** Mean scores (SD) for bvFTD and AD patients on demographics, behaviour and general cognitive tests.

Demographics, cognitive & behavioural tests	bvFTD	AD	F-test (p values)
N	30	18	
Age	61.6 (9.6)	64.1 (7.9)	0.3
Sex (M/F)	24/6	13/5	0.4
Education	11.88 (3.4)	12.86 (2.8)	0.3
Disease severity (Rasch score)	-0.81 (1.5)	0.75 (0.9)	*
CBI - total score	71.52 (28.8)	44.17 (26.8)	**
ACE -R (max. score = 100)	73.48 (12.8)	66.67 (24.6)	0.2
MMSE (max. score = 30)	24.52 (4.6)	22.17 (7.5)	0.2

* p<0.05;

** p<0.01;

*** p<0.001; CBI: Cambridge Behavioural Inventory; ACE-R: Addenbrooke's Cognitive Examination - Revised; MMSE: Mini-Mental State Examination.

**Behavioural Disturbances - Patients' Self-assessment**. Repeated measures ANOVA employing disease onset (before vs. after disease onset assessment), symptom (apathy vs. disinhibition vs. dysexecutive function) and diagnosis (bvFTD vs. AD) revealed a three-way interaction [F(1.72,43.04)=148.07, p<0.01]. Follow-up post-hoc tests split for diagnosis showed that AD patients themselves reported a change of behaviour from before to after the disease onset [F(1.6, 16.1)=238.4, p<0.01] and that changes were due to mostly dysexecutive functioning [t(10)=3.61, p<0.01] but not disinhibition (p>0.1) with a statistical trend for apathy (p=0.06). By contrast, bvFTDs' self-assessment reported no significant differences in behaviour from before to after disease onset (Table 2).

**Table 2:** Mean scores for bv-FTD and AD patients of the FrSBe (SD in brackets).

FrSBe		bvFTD	AD
**Patient self-assessment**			
Before disease	Apathy	70.6 (23)	61.6 (10)
	Disinhibition	73.7 (29)	61.1 (14)
	Executive dysfunction	65.2 (19)	62.2 (19)
After disease	Apathy	75.56 (21)	75.50 (18)*
	Disinhibition	80.69 (24)	69.17 (16)
	Executive dysfunction	71.63 (18)	**81.25 (18)**
**Carer assessment**			
Before disease	Apathy	71.8 (9)	66.3 (13)
	Disinhibition	65.5 (9)	65.4 (12)
	Executive dysfunction	63.2 (7)	61 (10)
After disease	Apathy	**87.55 (13)**	**77.21 (13)**
	Disinhibition	**77.69 (17)**	71.11 (16)
	Executive dysfunction	**79.03 (14)**	**77.42 (12)**

Bold indicates a significant change from before to after disease onset.

* indicates a statistical trend.

**Behavioural Disturbances - Carers' Assessment**. Analysis of carer assessments via repeated measure ANOVAs revealed an interaction of disease onset and symptom [F(1.7, 81.5)=335.3, p<0.01]. Follow-up tests revealed that AD carers corroborated the AD patients self-assessment via an interaction of disease onset by symptom [F(1.7, 31.4)=310.5, p<0.01]. Nevertheless, significant changes by the carers were not only observed for dysexecutive function [t(18)=5.96, p<0.001] but also apathy [t(18)=3.81, p<0.01] though not disinhibition (p>0.08). Similarly, carers of bvFTD patients reported a significant change pre- and post-disease onset [F(1, 48.8)=9284.44, p<0.001] across all behaviours (p's<0.001 for dysexecutive, apathy and disinhibition) (Table 2).

**Scan ratings**. The MRI scan ratings showed significant differences for atrophy of the total PFC (F(1, 42)=13.12, p<0.001) across groups (bvFTD AD) (Table 3). Follow-up analyses showed significant group effects (p's<0.001) for atrophy in all three PFC sub-regions (OFC, MFC, DLPFC), confirming the observation that bvFTD showed more atrophy overall in the PFC (Table 3).

**Table 3:** Mean scores (SD) for bv-FTD and AD patients in atrophy from visual rating scale.

MR visual ratings	bvFTD	AD	F-test
OFC	1.48 (1.3)	0.36 (0.7)	**
MPFC	2.12 (1.1)	1.19 (0.8)	**
DLPFC	2.30 (0.9)	1.42 (0.8)	**
Total PFC	11.80 (6.0)	5.94 (3.9)	**

** p<0.01; OFC: orbitofrontal cortex; MPFC: mesial prefrontal cortex; DLPFC: dorsolateral prefrontal cortex; PFC: prefrontal cortex.

Correlation analysis was performed on the scan ratings against the behavioural symptoms. A significant correlation was found between the OFC and apathy (r=0.376, p<0.025), as well as the MFC and apathy (r=0.344, p<0.025). No other significant correlations were found.

## DISCUSSION

Our study showed that bvFTD and AD patients and their carers differed for behavioural assessments on the FrSBe, with bvFTD patients showing little insight into their behavioural dysfunction, while AD patients and carers showed more similar behavioural change evaluations. Atrophy ratings showed that bvFTD patients had gross PFC atrophy in comparison to AD, which correlated for apathetic behaviours with atrophy in the medial and orbital frontal regions.

In more detail, contrasts of carers and patients assessment of behavioural changes in the patients showed a clear dissociation between the diagnoses. AD carer and patients agreeing that there has been a changed from before the disease onset, with both (carers and patients) concurring that dysexecutive functioning was the main problem. The carers reported further changes in motivation (i.e. apathy) which did not reach significance for the patient evaluations. For the bvFTD groups, carer and patients disagreed significantly on a change from before to after disease onset, with bvFTD patients rating themselves as behaviourally unchanged to before the disease. By contrast, carers of bvFTD patients rated all behavioural symptoms measures in FrSBe as significantly increased. These behavioural finding corroborate the well-known fact that bvFTD patients present with significantly more behavioural symptoms than AD patients.^24^ Further, our atrophy ratings corroborate previous findings by showing that bvFTD patients have more prefrontal cortex damage [23], though it should be noted that also the AD patients revealed prefrontal cortex atrophy but to a milder degree.

The discrepancy between behavioural assessment of the bvFTD carers and patients reflects again the pervasive insight issues this patient group has. It is striking that despite gross PFC atrophy and clear behavioural features the patients consider themselves not different than to before the disease started. Notably, both patients groups differ most significantly for the OFC ratings, which makes the OFC therefore a potential candidate for insight processing. Indeed, a previous study by Ruby and colleagues^25^ showed that insight into the disease covaried with grey matter atrophy in the OFC and functional neuroimaging studies have shown that self-evaluation in the healthy activated the OFC consistently.^26^

More importantly, our results show that atrophy in ventromedial prefrontal cortex regions (i.e. OFC, MFC) is linked to behavioural dysfunction linked to motivation. Other studies have attributed ventromedial dysfunction with disinhibition [e.g. 9], however there is mounting evidence that this region is also implicated in motivational dysfunction (i.e. apathy). For example, previous studies have used more sophisticated techniques, such as voxel-based morphometry, to identify the neural correlates of apathy and have reported similar regions. For example, Zamboni et al.^27^ showed that atrophy in OFC/MFC covaried with the apathy score on the FrSBe. Similarly, VBM^8^ and FDG-PET findings^9^ employing NPI scores showed that apathy was related to atrophy in right medial-orbital frontal brain regions using voxel-based morphometry. Our results corroborate these findings, however, we show for the first time that even a simple visual rating scale can detect such functional-anatomical correlates reliably. In the future, it would be interesting to investigate why the same region (i.e. ventromedial prefrontal cortex) is implicated in both disinhibition and apathy.^9^

Clinically, our study has shown that employment of questionnaires which take into account patient and carer evaluations have the benefit of establishing insight and behavioural symptoms at the same time. Further, the employment of our visual rating scale to relate the apathy findings to OFC and MFC atrophy may be useful as a diagnostic tool for clinicians. Lastly, our study has also found that carers and patients recognise different behavioural disturbances and physicians should therefore be aware to elicit information from both carers and patients as they may present contradicting symptoms with the same underlying disease. Still, our study only employed the first assessment of the behavioural symptoms and it would be interesting to know how behavioural disturbances change due to disease progression and severity in both conditions. Finally, replication of the FrSBe result using a different behavioural assessment tool or independent validation may further establish creditability of our results.
